# Correspondence: Reassessing the contribution of natural gas to US CO_2_ emission reductions since 2007

**DOI:** 10.1038/ncomms10648

**Published:** 2016-03-18

**Authors:** Matthew J. Kotchen, Erin T. Mansur

**Affiliations:** 1 School of Forestry and Environmental Studies, Yale University, New Haven, Connecticut 06511, USA; 2 National Bureau of Economic Research (NBER), Cambridge, Massachusetts 02138, USA; 3 Tuck School of Business, Dartmouth College, Hanover, New Hampshire 03755, USA

Energy-related CO_2_ emissions in the United States declined >10% between 2007 and 2013 (ref. 1), and understanding why is important for evaluating the prospects of existing and future US commitments to reduce emissions. In a recent paper, Feng *et al*.^2^ examined the drivers of US emissions^2^. Their paper has received a substantial amount of attention because of its conclusion that increased use of natural gas for electricity generation has contributed relatively little to the lowering of emissions. Here we offer an alternative interpretation of the Feng *et al*.^2^ analysis that supports the opposite conclusion. We argue that their results underscore the remarkable contribution natural gas has made to lowering emissions, and we offer two alternative methods for deriving comparable estimates.

Feng *et al*.^2^ estimated how different factors have caused changes in US emissions since 1997, with a particular focus on the decline from 2007 to 2013. They considered changes in the following six factors: population, consumption per capita, energy intensity, mix of consumption goods, mix of production inputs and fuel mix of the energy sector. Their main conclusion, as summarized in the abstract, is that ‘after 2007 decreasing emissions were largely a result of economic recession with changes in fuel mix (for example, substitution of natural gas for coal) playing a comparatively minor role'.

Their conclusion is based on interpretation of a waterfall chart (Fig. 3 of Feng *et al*.^2^) that shows the effect of each factor over three distinct intervals, 2007–2009, 2009–2011 and 2011–2013. The vast majority of emission reductions—totalling 9.9%—occurred from 2007 to 2009, and Feng, Davis, Sun, and Hubacek (FDSH) attribute more than half of this to the recession. Emissions remain relatively stable after 2009, and no other factor causes a comparable percentage change in emissions. This leads them to conclude further that ‘contrary to conventional wisdom, our decomposition analysis shows that changes in the fuel mix of the energy sector (including those related to the shale gas boom) account for a relatively small portion of this decrease'.

However, we argue that focusing on two-year intervals and emphasizing percentage changes in emissions provides a misleading picture about the relative importance of these drivers. Each driver has a cumulative effect over time; notably, recessions are always followed by periods of recovery, and it is not a viable nor advisable policy to rely on future recessions to reduce emissions. In our view, the important observation to explain in the data is not that the recession caused a significant decline in emissions, but rather that emissions have not climbed back to near pre-recession levels by 2013 despite the recovery.

To provide a more complete picture, we produce a comparable waterfall chart (Fig. 1) over the entire 2007–2013 interval using the Feng *et al*.^2^ results. Figure 1 illustrates how changes in the fuel mix caused a 4.4% decrease in emissions, and this exceeds the net decrease of 3.9% from the recession and subsequent recovery. However, neither of these drivers account for the largest drop in emissions during this time period. The authors find a striking 6.1% drop in total emissions due to changes in the production structure (that is, the mix of inputs, including domestic and imported materials), and this impressive effect deserves further exploration.

Figure 1 also illustrates how the total reduction in emissions is shared among drivers in percentage terms. In particular, changes in the fuel mix are responsible for 28.8% of the energy-related CO_2_ emission reductions. Rather than a ‘comparatively minor role', we interpret the authors' own findings as showing that changes in the fuel mix are responsible for a significant reduction in CO_2_ emissions from 2007 to 2013.

The Feng *et al*.^2^ methodological approach has the advantage of generating counterfactuals, from which they can estimate the partial effect (positive or negative) of each factor on emissions. There are, however, more direct and transparent methods to specifically estimate the contribution of substituting natural gas for coal in electricity generation. Here we briefly describe two approaches and use the results as external validity tests of the Feng *et al*.^2^ analysis and our interpretation.

The first method examines changes over time in the average emission rate of all electricity generated from coal and natural gas, using the Energy Information Administration (EIA) data on annual emissions^3^ and generation^4^. This rate has been mostly in decline since 2007 because of greater substitution of natural gas for coal. We predict what annual emissions would have been without substitution to more natural gas by simply taking the product of the 2007 emission rate and the combined electricity generation in each subsequent year. Figure 2 plots the percent change in emissions from 2007 to each subsequent year using this approach. We consider three different bases for purposes of comparison: annual emissions from all fossil-fuel generated electricity^5^, all energy-related emissions^1^ and US emissions from all sources^5^. The figure shows how the reductions begin to occur in 2009 when the price of natural gas declines significantly. By 2012 and 2013, fuel switching is responsible for reducing emissions from electricity generation between 7.3 and 8.9%, and from all sources of US emissions between 2.9 and 3.6%. As a share of the overall reduction of emissions from 2007 to 2013, this method attributes 28.2% to greater use of natural gas in electricity generation.

The second method for estimating the contribution of natural gas to emission reductions is based on the relationship between fuel prices and emissions from the electricity sector. Cullen and Mansur^6^ estimate the relationship using the ratio of coal to natural gas prices in a regression model that controls for electricity consumption, temperature, generation from non-fossil sources, net imports of electricity from Canada and seasonal effects^6^. We use the coefficients from their model, along with Henry Hub natural gas prices and Central Appalachia coal prices^7^,^8^ (from January 2008 to December 2013), to predict changes in daily emissions relative to a 2007 baseline and derive annual emission reductions for each year. While this approach is less transparent than the one described previously, it has the advantage over our first approach of not assuming a simple linear relationship between emissions and electricity generation. Instead, the regression approach uses information about how emissions actually changed over time to separately identify the effect of fuel prices from other factors, including energy demand and renewables investment. Nevertheless, we find similar results when looking at how the relative drop in natural gas prices affected emissions. We found that fuel switching to natural gas for electricity generation is responsible for reducing overall US emissions between 2.1 and 4.3%. These point estimates imply that the share of emissions reductions that is due to low natural gas prices is between 20.4 and 40.7%—a range that includes the estimates implied by FDSH's analysis and those from our previous approach.

Understanding the role natural gas has played in lowering US CO_2_ emissions is important for evaluating the ongoing impacts of US climate policy and international commitments as part of the United Nations climate agreement in Paris. FDSH make an important contribution by estimating the relative magnitudes of different drivers of US emissions. We find their analysis compelling, yet draw a different conclusion about the importance of natural gas. Rather than playing a relatively minor role, we have argued that their analysis shows how the shale gas revolution has played a significant role, accounting for 29% of US emission reductions between 2007 and 2013. Alternative estimates that we provide attribute up to 40% of the emission reductions to natural gas. Hence, instead of challenging conventional wisdom about the importance of natural gas to US emissions and climate policy, FDSH's results are closely in line with those from alternative approaches that we describe here and others that are referenced in official government reports^9^,^10^.

## Additional information

**How to cite this article**: Kotchen M. J. & Mansur E. T. Correspondence: Reassessing the contribution of natural gas to US CO_2_ emission reductions since 2007. *Nat. Commun*. 7:10648 doi: 10.1038/ncomms10648 (2016).

## Figures and Tables

**Figure 1:**
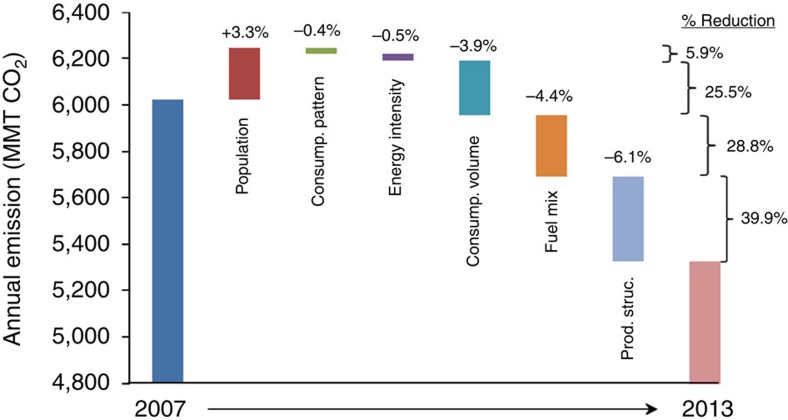
Contributions of different factors to the decline of US CO_2_ emissions 2007–2013. Numbers are taken from FDSH's structural decomposition analysis that spans the whole time period. Changes in the fuel mix are responsible for a 4.4% decrease in energy-related emissions and 28.8% of the emissions decreases that occurred during this period. This amount exceeds that from changes in consumption volume (Consump. volume), but is less than that from changes in production structure (Prod. struct.).

**Figure 2:**
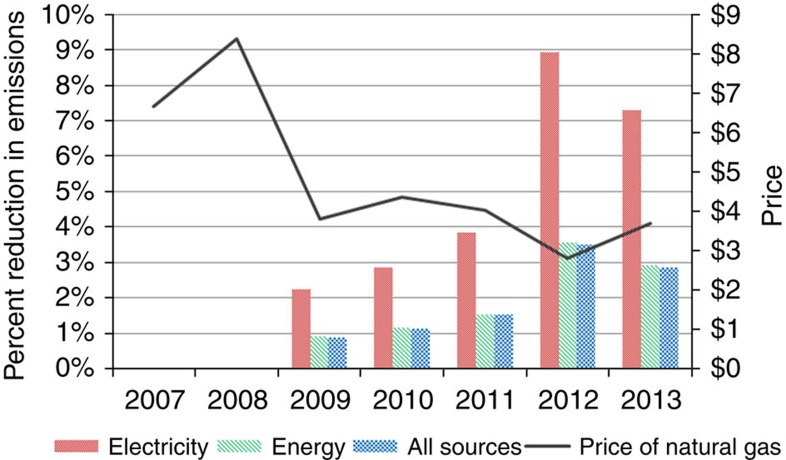
Annual percentage reduction of US CO_2_ emissions compared with 2007 levels due to fuel switching of natural gas for coal in electricity generation and annual natural gas prices. Forecasts are based on holding the emission rate from coal and natural gas constant at the 2007 level. Bases for comparison are annual emissions from all fossil-fuel generated electricity, all energy-related emissions and US emissions from all sources. As a share of the overall reduction of emissions from 2007 to 2013 of 10.3%, this method attributes 28.2% to greater use of natural gas in electricity generation.
